# Dapsone‐ and nitroso dapsone‐specific activation of T cells from hypersensitive patients expressing the risk allele HLA‐B*13:01

**DOI:** 10.1111/all.13769

**Published:** 2019-04-15

**Authors:** Qing Zhao, Khetam Alhilali, Abdulaziz Alzahrani, Mubarak Almutairi, Juwaria Amjad, Hong Liu, Yonghu Sun, Lele Sun, Huimin Zhang, Xiaoli Meng, Andrew Gibson, Monday O. Ogese, B. Kevin Park, Jianjun Liu, David A. Ostrov, Furen Zhang, Dean J. Naisbitt

**Affiliations:** ^1^ MRC Centre for Drug Safety Science, Department of Molecular and Clinical Pharmacology The University of Liverpool Liverpool UK; ^2^ Department of Dermatology, Shandong Provincial Hospital for Skin Disease Shandong University Jinan China; ^3^ Shandong Provincial Institute of Dermatology and Venereology Shandong Academy of Medical Sciences Jinan China; ^4^ Al Baha University, Prince Mohammad Bin Saud Al Bahah Saudi Arabia; ^5^ Pharmacology Department, College of Clinical Pharmacy AlBaha University Al Baha Saudi Arabia; ^6^ Pathological Sciences, drug Safety and Metabolism, IMED Biotech Unit AstraZeneca Cambridge UK; ^7^ Human Genetics Genome Institute of Singapore, A*STAR Singapore Singapore; ^8^ Department of Pathology, Immunology and Laboratory Medicine College of Medicine University of Florida Gainesville Florida

**Keywords:** drug hypersensitivity, human, T cells

## Abstract

**Background:**

Research into drug hypersensitivity associated with the expression of specific HLA alleles has focussed on the interaction between parent drug and the HLA with no attention given to reactive metabolites. For this reason, we have studied HLA‐B*13:01‐linked dapsone hypersensitivity to (a) explore whether the parent drug and/or nitroso metabolite activate T cells and (b) determine whether HLA‐B*13:01 is involved in the response.

**Methods:**

Peripheral blood mononuclear cells (PBMC) from six patients were cultured with dapsone and nitroso dapsone, and proliferative responses and IFN‐γ release were measured. Dapsone‐ and nitroso dapsone‐specific T‐cell clones were generated and phenotype, function, HLA allele restriction, and cross‐reactivity assessed. Dapsone intermediates were characterized by mass spectrometry.

**Results:**

Peripheral blood mononuclear cells from six patients and cloned T cells proliferated and secreted Th1/2/22 cytokines when stimulated with dapsone (clones: n = 395; 80% CD4^+^ CXCR3^hi^CCR4^hi^, 20% CD8+CXCR3^hi^CCR4^hi^CCR6^hi^CCR9^hi^CCR10^hi^) and nitroso dapsone (clones: n = 399; 78% CD4+, 22% CD8^+^ with same chemokine receptor profile). CD4^+^ and CD8^+^ clones were HLA class II and class I restricted, respectively, and displayed three patterns of reactivity: compound specific, weakly cross‐reactive, and strongly cross‐reactive. Nitroso dapsone formed dimers in culture and was reduced to dapsone, providing a rationale for the cross‐reactivity. T‐cell responses to nitroso dapsone were dependent on the formation of a cysteine‐modified protein adduct, while dapsone interacted in a labile manner with antigen‐presenting cells. CD8^+^ clones displayed an HLA‐B*13:01‐restricted pattern of activation.

**Conclusion:**

These studies describe the phenotype and function of dapsone‐ and nitroso dapsone‐responsive CD4^+^ and CD8^+^ T cells from hypersensitive patients. Discovery of HLA‐B*13:01‐restricted CD8^+^ T‐cell responses indicates that drugs and their reactive metabolites participate in HLA allele‐linked forms of hypersensitivity.

**Abbreviations**
APC: antigen‐presenting cells
DDS: dapsone
DDS‐NO: nitroso dapsone
PBMC: peripheral blood mononuclear cells
SI: stimulation index

## 1 INTRODUCTION

Genome‐wide association studies have identified strong associations between the expression of HLA alleles and susceptibility to drug hypersensitivity.^1^ These data suggest that drugs bind selectively to the HLA protein to activate the T cells that participate in the adverse event. Molecular docking studies support this concept^2^, ^3^; however, modeling data have to be interpreted with caution as the nature of the drug‐HLA protein interaction and the requirement for a specific peptide in the binding groove has not been determined. The most robust genetic associations are between HLA class I alleles and abacavir hypersensitivity,^4^ flucloxacillin liver injury (both HLA‐B*57:01),^5^ carbamazepine‐induced Stevens Johnson syndrome (HLA‐B*15:02),^6^ and allopurinol hypersensitivity (HLA‐B*58:01),^7^ and in each case, mechanistic studies have shown that CD8^+^ T cells are activated when the drug interacts with the relevant HLA protein.^7^, ^8^ For abacavir and carbamazepine, the drug HLA binding site is very different; abacavir binds deep in the HLA peptide binding pocket, while carbamazepine binds to a site closer to the T‐cell receptor interface. Despite this, both drugs interact with HLA proteins via a reversible interaction to stimulate T cells. T cells from patients with allopurinol hypersensitivity are activated with a stable metabolite, oxypurinol, also via a direct binding interaction with HLA. In contrast, flucloxacillin‐specific T cells from patients with liver injury are activated with drug‐protein adducts, via a hapten mechanism involving the spontaneous binding of the drug to protein and antigen processing. This brief discussion illustrates that rapid progress has been made in our understanding of the relationship between drug HLA binding and the activation of T cells; however, reactive drug metabolites have been ignored in the study of HLA allele‐restricted forms of drug hypersensitivity. This is primarily because of the absence of synthetic reactive metabolites for functional studies with T cells from hypersensitive patients.

A reactive metabolite of sulfamethoxazole is known to activate patient T cells via a hapten mechanism^12^, ^13^; however, sulfamethoxazole reactions are not associated with a specific HLA allele. Thus, we recently synthesized the nitroso metabolite of dapsone and studied the priming of naïve T cells from healthy donors.^15^ Dapsone contains a sulfone group that links two aromatic amine moieties. Oxidative metabolism of the amine groups generates a hydroxylamine. The hydroxylamine undergoes spontaneous oxidation to form nitroso dapsone, which binds covalently to cellular proteins.^16^, ^17^ Naïve CD4^+^ and CD8^+^ T cells from healthy donors are activated with the parent drug and nitroso metabolite when Tregs were removed and the compounds were presented by dendritic cells.^15^ These data show that both forms of the drug interact with multiple HLA molecules and have the capacity to stimulate T cells when regulatory pathways have been manipulated.

Dapsone is used in combination with other drugs for the treatment of infectious diseases such as leprosy and malaria. 0.5%‐3.6% of treated patients develop a hypersensitivity syndrome characterized by fever, skin rash, and internal organ involvement 4‐6 weeks after treatment commences.^18^ HLA‐B*13:01 is associated with the development of dapsone hypersensitivity in Chinese and Thai patients,^19^, ^20^ and modeling data suggest that dapsone may fit in the peptide recognition site of HLA‐B*13:01.^21^ Dapsone‐treated cell lines expressing HLA‐B*13:01 have been shown to activate T cells, while peripheral blood mononuclear cells (PBMC) from 2/7 patients secrete high levels of the cytolytic molecule granulysin when stimulated with the drug.^22^ Despite this, a detailed analysis of the phenotype and function of dapsone‐specific T cells has not been performed. Moreover, the activation of patient T cells with nitroso dapsone has not been investigated. Thus, our study had three primary objectives: to investigate whether dapsone and/or nitroso dapsone activates CD4^+^ and CD8^+^ T cells from hypersensitive patients, to define phenotype and function of drug‐specific T cells, and to explore whether HLA‐B*13:01 is directly involved in the drug‐/drug metabolite‐specific T‐cell response.

## 2 METHODS

### 2.1 Human subjects

Venous blood (50 mL) was collected from 6 dapsone hypersensitive patients. Table 1 summarizes the demographics of the patients. Table S1 shows results of HLA typing. HLA‐B*13:01+ donors (n = 4) with no history of dapsone exposure and HLA‐B*13:01+ dapsone‐tolerant patients (n = 4) were selected as a control groups. Patch testing was conducted on the back of patients with dapsone (0.1%‐25%). The patch was removed after 48 hours. Results were recorded after a further 24 hours to exclude any false‐positive responses resulting from the patch tape. The study was approved by the Ethical Committee of the Shandong Provincial Institute of Dermatology and Venereology, and informed written consent was obtained. A material transfer agreement was signed prior to shipment of PBMC to Liverpool.

**Table 1:** Patients’ demographics and details of the hypersensitivity reaction

Patient ID	Gender	Age (years)	Medication history	Onset of symptoms (days)	Clinical presentation	Skin patch test
1	Female	43	Dapsone, rifampin, and clofazimine	3	Fever, rash, and abnormal liver function tests	−
3	Female	25	Dapsone, rifampin, and clofazimine	28	Fever, rash, and abnormal liver function tests	−
5	Male	39	Dapsone, rifampin, and clofazimine	30	Fever	+
6	Male	41	Dapsone, rifampin, and clofazimine.	48	Fever, rash, and lymphadenopathy	+
7	Male	54	Dapsone, rifampin, and clofazimine	16	Fever, rash, and abnormal liver function tests (AST 47.7 U/L; ALP132.3 U/L)	−
8	Female	27	Dapsone, rifampin, and clofazimine	17	Fever and abnormal liver function tests (AST 56.3 U/L; ALP 225.7 U/L; GGT 184.8 U/L; TBIL 38.8 μmol/L; DBIL 21.3 μmol/L; IBIL 17.5)	+

### 2.2 Lymphocyte transformation test and PBMC ELIspot

Peripheral blood mononuclear cells (1.5 × 10^5^ cell/well) from hypersensitive patients and control donors were incubated with dapsone (125‐500 µmol/L), nitroso dapsone (10‐40 µmol/L), rifampicin (10‐100 µmol/L), clofazimine (10‐100 µmol/L), or tetanus toxoid (5 μg/mL, as a positive control) in culture medium for 5 days. [^3^H]thymidine was added for the final 16 hours of the experiment. IFN‐γ‐secreting PBMC were visualized using ELIspot by culturing PBMC (5 × 10^5 ^cell/well) in medium with/without optimal concentrations of dapsone or nitroso dapsone for 48 hours.

### 2.3 Generation and characterization of drug‐specific T‐cell clones

Dapsone and nitroso dapsone‐responsive T‐cell clones were generated from patients 5, 6, and 8 by serial dilution and characterized in terms of cellular phenotype (CD, TCR Vβ, and chemokine receptors [flow cytometry]), secretion of cytokines and cytolytic molecules (ELIspot), antigen specificity (dose‐response studies and cross‐reactivity with structurally‐related compounds [^3^H]thymidine uptake]), pathways of activation (antigen‐presenting cell pulsing experiments, fixation of antigen‐presenting cells to block processing, and addition of glutathione to block drug metabolite protein binding), and the involvement of HLA‐B*13:01 in the T‐cell response (HLA antibody blocking experiments and use of partly HLA matched antigen‐presenting cells). Detailed methods are available in as Data S1.

### 2.4 Statistics

All statistical analysis (one‐way ANOVA unless stated otherwise) was performed using SigmaPlot 12 software (*P < 0.05).

## 3 RESULTS

### 3.1 Patch testing and in vitro activation of hypersensitive patient PBMC

**Figure 1:**
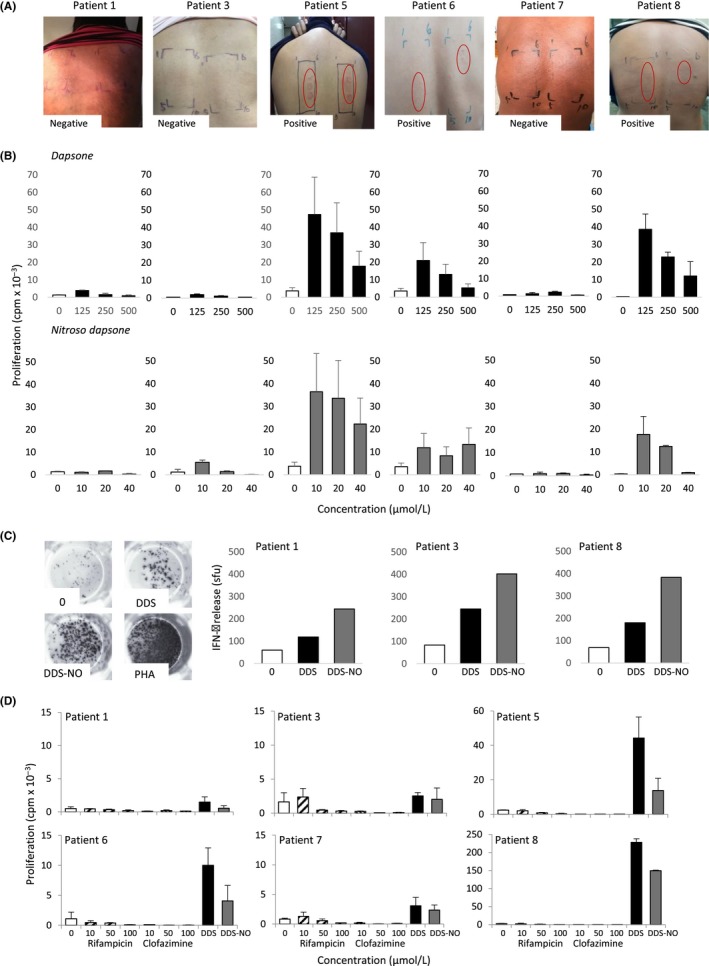
Diagnosis of dapsone hypersensitivity by skin testing and in vitro assays. A, Skin patch test results for dapsone hypersensitivity. Skin was exposed to dapsone in polyethylene glycol 200 at dilutions of 0%, 0.1%, 0.5%, 1%, 5%, 10%, 15%, 2%, and 25%. The patch tape was left to the skin for 48 h, and diagnosis was made 24 h later. Red ovals show areas of inflamed skin. B, Peripheral blood mononuclear cells (PBMC) from patients were exposed to graded concentrations of dapsone or nitroso dapsone. Proliferation was measured after 5 d by the addition of [^3^H]thymidine for 16 h. Results are expressed as mean ± SD cpm of triplicate cultures. A doubling of cpm in drug‐treated cultures over vehicle control is considered positive. C, PBMC from patients were exposed to optimal concentrations of dapsone or nitroso dapsone, and IFN‐γ release was visualized by ELIspot. D, PBMC from patients were exposed to graded concentrations of dapsone, nitroso dapsone, rifampicin, and clofazimine. Proliferation was measured as described in (B) (DDS, dapsone; DDS‐NO, nitroso dapsone; PHA, phytohaemagglutinin)

After 72‐hour drug exposure, 3 out of 6 patients displayed dapsone concentration‐dependent positive readings (Figure 1A). PBMC from all 3 patch test‐positive patients were stimulated to proliferate strongly in the presence of dapsone and nitroso dapsone (Figure 1B). Positive dapsone‐specific proliferative responses (stimulation index [SI] 2 or above) and/or IFN‐γ secretion were also detected with PBMC from the 3 patch test‐negative patients, while nitroso dapsone responses were detected in 2 patients (Figure 1B‐D). Of note, patient 7 displayed a negative nitroso dapsone lymphocyte transformation test response on initial testing (Figure 1B) and a weak response when the assay was repeated (Figure 1D). PBMC were not activated with the co‐medications rifampicin and clofazimine (Figure 1D). PBMC from dapsone‐naïve (SI < 2) and dapsone‐tolerant (Figure S1) HLA‐B*13:01+ controls proliferated in the presence of phytohemagglutinin, but not the test drugs.

### 3.2 Dapsone and nitroso dapsone activate CD4^+^ and CD8^+^ clones

A total of 1334 and 1374 CD4^+^ and CD8^+^ clones, respectively, were expanded from dapsone and nitroso dapsone T‐cell lines from patients 5, 6, and 8; 626 CD4^+^ clones displayed reactivity against dapsone or nitroso dapsone (Figure S2). Dapsone‐ and nitroso dapsone‐specific CD8^+^ clones were generated in lower numbers; 168 were activated with either the drug or drug metabolite. Dapsone‐ and nitroso dapsone‐responsive CD4^+^ and CD8^+^ clones were detected in equal numbers (Table 2).

**Table 2:** Phenotype and drug specificity of T‐cell clones generated from dapsone hypersensitive patients

Phenotype and drug specificity	Total number of clones	Number of drug‐specific clones	Percentage of responding clones (%)
Patient 8			
CD4^+^, DDS^+^	236	176	74.58
CD8^+^, DDS^+^	245	29	11.84
CD4^+^, DDS‐NO^+^	384	210	54.69
CD8^+^, DDS‐NO^+^	304	54	17.76
Patient 6			
CD4^+^, DDS^+^	177	76	42.94
CD8^+^, DDS^+^	178	14	7.87
CD4^+^, DDS‐NO^+^	208	57	27.40
CD8^+^, DDS‐NO^+^	285	15	5.26
Patient 5			
CD4^+^, DDS^+^	180	64	35.56
CD8^+^, DDS^+^	170	36	21.18
CD4^+^, DDS‐NO^+^	149	43	28.86
CD8^+^, DDS‐NO^+^	192	20	10.42

**Figure 2:**
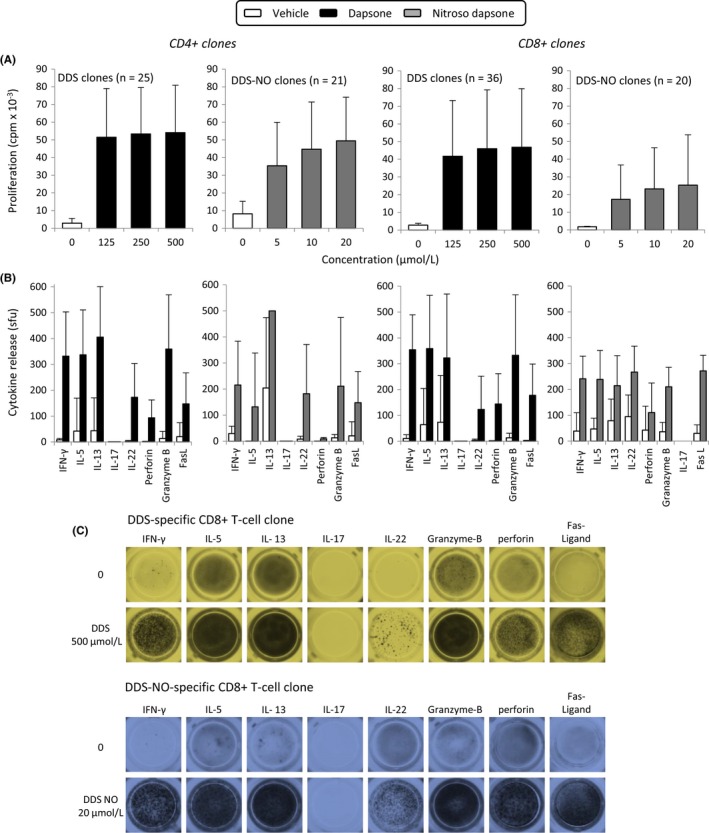
Proliferative response and cytokine release by dapsone‐ and nitroso dapsone‐responsive CD4^+^ and CD8^+^ clones. A, One hundred and two CD4^+^ or CD8^+^ clones were incubated with autologous antigen‐presenting cells and either dapsone (125‐500 µmol/L) or nitroso dapsone (5‐20 µmol/L) in triplicate cultures for 48 h. Proliferative responses were measured by the addition of [^3^H]thymidine. Results are expressed as mean ± SD cpm of the indicated number of clones. B, Detection of IFN‐γ, IL‐5, IL‐13, IL‐17, IL‐22, granzyme B, perforin, and Fas‐ligand secretion by dapsone‐ and nitroso dapsone‐responsive CD4^+^ and CD8^+^ clones. Clones were incubated with autologous antigen‐presenting cells, and either dapsone or nitroso dapsone and cytokine release was visualized by ELIspot. C, Representative ELIspot images showing the cytokines released by dapsone and nitroso dapsone‐responsive CD8^+^ clones

One hundred and two well‐growing clones were selected for dose‐titration studies and the analysis of cytokine secretion. Proliferative responses were detected with 3 well‐tolerated concentrations of dapsone (125‐500 µmol/L) and nitroso dapsone (5‐20 µmol/L) and drug treatment resulted in the secretion of Th1 (IFNγ), Th2 (IL‐5, IL‐13), and Th22 (IL‐22) cytokines, alongside the cytolytic molecules perforin, granzyme B, and FasL. CD4^+^ and CD8^+^ clones secreted similar levels of cytokines and cytolytic molecules (Figure 2). Table S3 shows the percentage of dapsone (DDS)‐ and nitroso dapsone (DDS‐NO)‐responsive CD4^+^ and CD8^+^ clones that secrete individual cytokines.

### 3.3 Dapsone‐ and nitroso dapsone‐responsive CD4^+^ and CD8^+^ clones display three distinct patterns of cross‐reactivity

**Figure 3:**
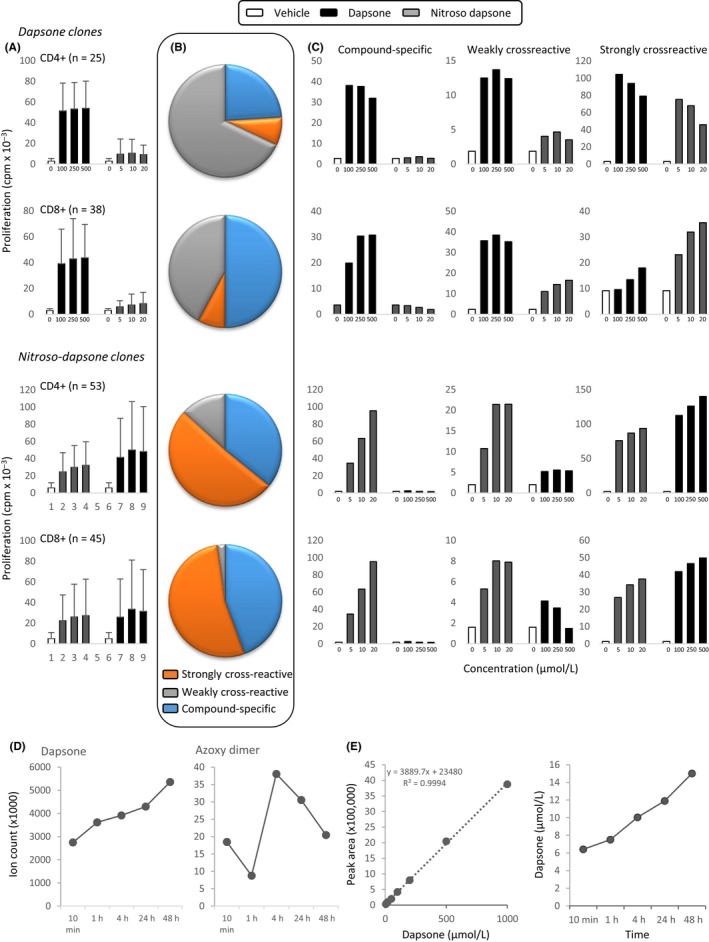
Cross‐reactivity of dapsone‐ and nitroso dapsone‐responsive T‐cell clones. Dapsone‐ and nitroso dapsone‐responsive CD4^+^ and CD8^+^ clones were cultured with irradiated autologous EBV‐transformed B cells and either dapsone (100‐500 µmol/L) or the nitroso metabolite (5‐20 µmol/L) in triplicate cultures for 48 h. T‐cell proliferative responses were assessed through the addition of [^3^H]‐thymidine. A, Mean cross‐reactivity data for 153 clones divided according to the drug antigen peripheral blood mononuclear cells were cultured with to generate clones and CD phenotype. B, Pie charts showing number of clones with a particular cross‐reactivity profile (compound specific, weekly cross‐reactive (cross‐reactive compound displaying 10%‐50% response detected with comparator) and strongly cross‐reactive (cross‐reactive compound displaying > 50% response detected with comparator). C, Representative clones displaying each response profile. D, Relative quantification of dapsone and azoxy dimer in cultures containing nitroso dapsone (30 µmol/L), autologous EBV‐transformed B cells, and T‐cell clones. E, Absolute quantification of dapsone formation in cultures containing nitroso dapsone (30 µmol/L), autologous EBV‐transformed B cells, and T‐cell clones. Left hand graph shows the standard curve for dapsone (concentration range: 5 nmol/L‐1 µmol/L). Right hand side graph shows the time‐dependent formation of dapsone [Colour figure can be viewed at wileyonlinelibrary.com]

Sixty‐three dapsone‐ and 98 nitroso dapsone‐responsive CD4^+^ and CD8^+^ clones were assayed for cross‐reactivity. When all of the dapsone‐responsive clones were assessed together, low levels of proliferation were observed with nitroso dapsone (Figure 3A). The maximum concentration of nitroso dapsone used was 10 times lower than the dapsone concentration. Analysis of individual clones revealed three distinct cross‐reactivity patterns: dapsone‐specific, and weakly and strongly cross‐reactive with nitroso dapsone. Approximately 90% of the clones were dapsone‐specific or weakly cross‐reactive (Figure 3B,C).

Nitroso dapsone CD4^+^ and CD8^+^ clones displayed a much higher level of cross‐reactivity (Figure 3A). However, 3 patterns of cross‐reactivity were again observed with individual clones: nitroso dapsone‐specific, and weakly and strongly cross‐reactive with dapsone. In contrast to the dapsone‐responsive clones, approximately 90% of the nitroso dapsone‐responsive clones were nitroso dapsone‐specific or highly cross‐reactive (Figure 3B,C).

Dapsone‐ and nitroso dapsone‐responsive clones were also stimulated to proliferate with dapsone hydroxylamine; however, proliferative responses were not detected when clones were cultured with (a) dapsone analogues with substitutions in the sulfone group, (b) dapsone analogues with amine groups in different positions on the aromatic rings, and (c) structurally distinct sulfonamide antimicrobials (Figure S3).

The stability of nitroso dapsone in the proliferation assay was assessed. Nitroso dapsone was converted rapidly to azoxy dimers and the parent compound. Both compounds were detectable within 10 minutes. After 2 days, 50% of nitroso dapsone had been converted to dapsone (Figure 3D‐E).

### 3.4 Cross‐reactive clones are activated with equivalent concentrations of dapsone and nitroso dapsone

**Figure 4:**
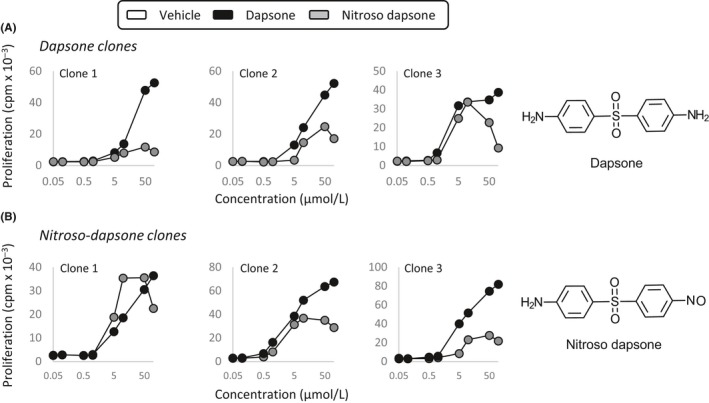
Dapsone and nitroso dapsone activate T‐cell clones at the same minimum concentration. A, Dapsone‐ and (B) nitroso dapsone‐responsive CD4^+^ and CD8^+^ clones were cultured with irradiated autologous EBV‐transformed B cells and either dapsone (0.1‐100 µmol/L) or the nitroso metabolite (0.1‐100 µmol/L) in triplicate cultures for 48 h. T‐cell proliferative responses were assessed through the addition of [^3^H]‐thymidine. Representative weakly and strongly cross‐reactive dapsone and nitroso dapsone‐responsive clones are shown

Six strong and weakly cross‐reactive clones were incubated with dapsone and nitroso dapsone at concentrations of 0.1‐100 µmol/L to define the minimum stimulatory concentrations of the two compounds. Clones were stimulated to proliferate with equivalent concentrations of each compound irrespective of the extent of cross‐reactivity (Figure 4).

### 3.5 Dapsone‐ and nitroso dapsone‐responsive CD4^+^ and CD8^+^ clones expressing multiple TCR Vβ chains display distinct chemokine receptor profiles

Dapsone‐ and nitroso dapsone‐responsive CD4^+^ and CD8^+^ clones expressed single, but variable TCR Vβ chains (Figure S4A), with no clear differences discernible when CD4^+^ and CD8^+^ or dapsone‐ and nitroso dapsone‐responsive clones were compared. Dapsone‐ and nitroso dapsone‐responsive CD4^+^ clones expressed high levels of the chemokine receptors CXCR3 and CCR4. The CD8^+^ clones expressed CXCR3 and CCR4 alongside CCR10, CCR9, and CCR6 (Figure S4B).

### 3.6 HLA‐restricted activation of dapsone‐ and nitroso dapsone‐responsive clones ensues via different mechanisms

**Figure 5:**
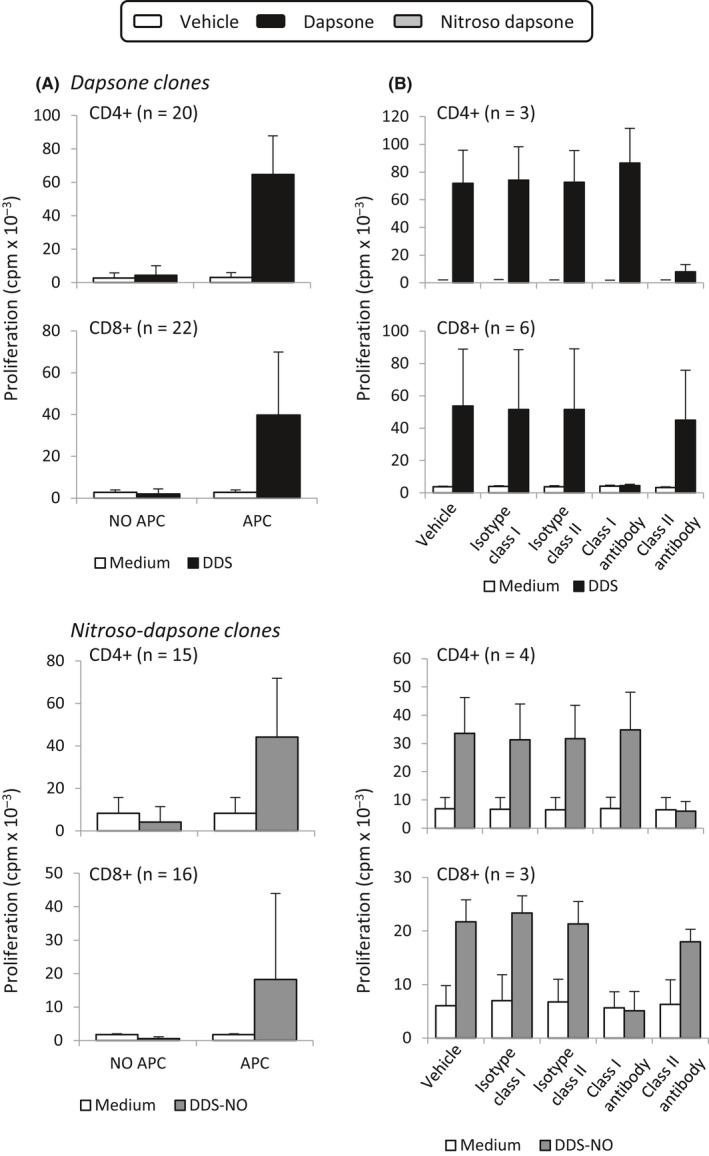
Antigen‐presenting cells are required for the activation of dapsone‐ and nitroso dapsone‐responsive CD4^+^ and CD8^+^ clones. A, Dapsone‐ and nitroso dapsone‐responsive CD4^+^ and CD8^+^ clones were cultured with dapsone (500 µmol/L) or the nitroso metabolite (20 µmol/L) in triplicate cultures for 48 h either in the presence or absence of autologous EBV‐transformed B cells. B, Dapsone‐ and nitroso dapsone‐responsive CD4^+^ and CD8^+^ clones were cultured with antigen‐presenting cells and dapsone (500 µmol/L) or the nitroso metabolite (20 µmol/L) in triplicate cultures for 48 h either in the presence or absence of anti‐HLA class I and II blocking antibodies. [^3^H]‐thymidine was added for 16 h to measure drug‐specific proliferative responses

**Figure 6:**
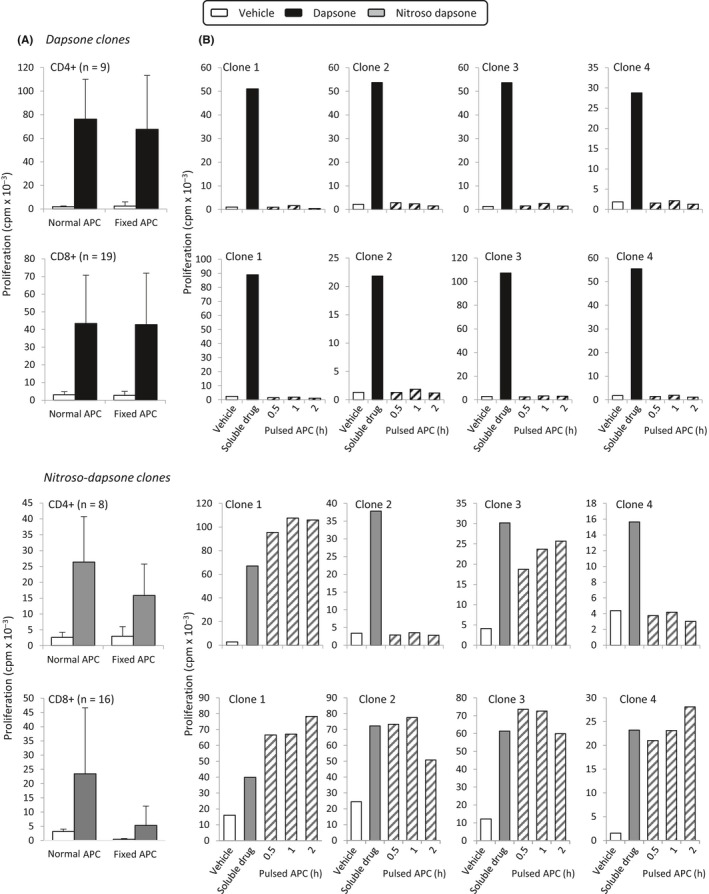
Dapsone and nitroso dapsone activate T cells via different pathways. A, Dapsone‐ and nitroso dapsone‐responsive CD4^+^ and CD8^+^ clones were cultured with dapsone or the nitroso metabolite in the presence of either irradiated or glutaraldehyde‐fixed autologous EBV‐transformed B cells (APC, antigen‐presenting cells) in triplicate cultures for 48 h. Fixation blocks antigen processing. B, Dapsone‐ and nitroso dapsone‐responsive CD4^+^ and CD8^+^ clones were cultured with drug‐ or drug metabolite‐pulsed (0.5‐2 h) irradiated autologous EBV‐transformed B cells in the absence of soluble drug in triplicate cultures for 48 h. T‐cell proliferative responses were assessed through the addition of [^3^H]‐thymidine

Stimulation of dapsone‐ and nitroso dapsone‐responsive CD4^+^ and CD8^+^ clones was dependent on the presence of antigen‐presenting cells (Figure 5A). Use of blocking antibodies revealed that CD4^+^ and CD8^+^ proliferative responses to dapsone and nitroso dapsone were HLA class II and I restricted, respectively (Figure 5B). Fixation of antigen‐presenting cells with glutaraldehyde had no effect on the activation of CD4^+^ or CD8^+^ clones with dapsone. In contrast, antigen‐presenting cell fixation reduced the extent of proliferation with the nitroso metabolite (Figure 6A). The residual response detected with nitroso dapsone and fixed antigen‐presenting cells relates to the conversion of nitroso dapsone to dapsone in culture.

Nitroso dapsone‐responsive CD8^+^ clones were activated with antigen‐presenting cells pulsed with nitroso dapsone for 0.5‐2 hours (Figure 6B). Two out of 4 nitroso dapsone‐responsive CD4^+^ clones were also stimulated to proliferate with nitroso dapsone‐pulsed antigen‐presenting cells. Antigen‐presenting cells pulsed with dapsone for 0.5‐2 hours did not activate the dapsone‐responsive CD4^+^ or CD8^+^ clones.

Figure S5A compares the level of nitroso dapsone glutathione adducts formed in the cell culture assay in the presence and absence of exogenous glutathione.^23^, ^24^ Adducts were formed rapidly (within 2 hours), and the level of adduct formation was several orders of magnitude higher in the presence of exogenous glutathione. Figure S6 contains traces showing how the adducts were quantified. The addition of glutathione to the T‐cell assay containing soluble nitroso dapsone reduced the strength of the proliferative response, while the response to nitroso dapsone‐pulsed antigen‐presenting cells was completely blocked (Figure S5B).

### 3.7 Dapsone and nitroso dapsone bind with a degree of selectively to HLA‐B*13:01 to activate certain CD8^+^ T‐cell clones

EBV‐transformed B cells were generated from 9 healthy donors expressing HLA alleles with >90% sequence homology to HLA‐B*13:01. HLA typing of the healthy donors is shown in Table S2. The B‐cell lines were used to explore the requirement for HLA‐B*13:01 in dapsone‐ and nitroso dapsone‐specific CD8^+^ T‐cell activation. Autologous antigen‐presenting cells and antigen presenting from 1 additional patients were used as comparators.

**Figure 7:**
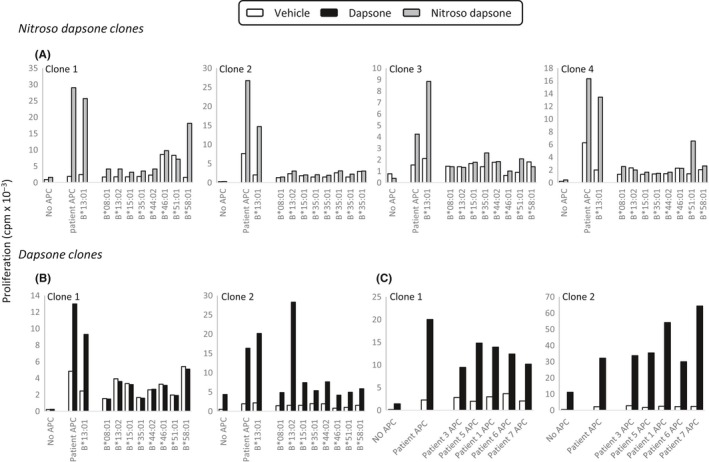
Dapsone and nitroso dapsone bind with a degree of selectively to HLA‐B*13:01 to activate certain CD8^+^ T‐cell clones. A, Nitroso dapsone‐ and (B) dapsone‐responsive CD8^+^ clones were cultured with drug or drug metabolite and irradiated EBV‐transformed B cells (APC, antigen‐presenting cells) from 10 donors expressing different HLA‐B alleles in triplicate cultures for 48 h. The complete HLA type of the different donors is shown in Table S2. C, Dapsone‐responsive CD8^+^ clones were cultured with dapsone and irradiated EBV‐transformed B cells from 6 hypersensitive patients expressing HLA‐B*13:01 in triplicate cultures for 48 h. T‐cell proliferative responses were assessed through the addition of [^3^H]‐thymidine

As has been described previously for other drugs,^25^, ^26^ 30% of dapsone and nitroso dapsone‐responsive HLA class I restricted CD8 clones displayed proliferative responses with the drug or metabolite and antigen‐presenting cells expressing a wide variety of HLA alleles (results not shown). Other clones were activated with the drug or metabolite only in the presence of autologous antigen‐presenting cells. Despite this, 30%‐40% of CD8^+^ clones displayed a degree of HLA‐B*13:01 allele restriction. Figure 7A,B shows 3 clones (1 dapsone‐ and 2 nitroso dapsone‐responsive) that were stimulated to proliferate exclusively in the presence of dapsone or nitroso dapsone and antigen‐presenting cells expressing HLA‐B*13:01. Three additional clones (1 dapsone‐ and 2 nitroso dapsone‐responsive) are shown that were activated in the presence of antigen‐presenting cells expressing HLA‐B*13:01 and either B*13:02, B*58:01, or B*51:01.

The 2 dapsone‐responsive CD8^+^ clones were expanded in sufficient numbers for us to conduct a proliferation assay using antigen‐presenting cells from the 6 hypersensitive patients that all express HLA‐B*13:01. The clones were activated in the presence of dapsone and antigen‐presenting cells from all of the patients (Figure 7C).

## 4 DISCUSSION

Knowledge of the role drug metabolism plays in the generation of antigenic determinants that activate T cells is limited. Circumstantial evidence supporting a role for drug metabolism includes the identification of protein‐reactive metabolites for many drugs associated with a high incidence of hypersensitivity and the induction of toxicity in target tissue by reactive metabolites and hence the potential to disrupt immune regulatory pathways through the provision of danger signals.^27^ However, the most direct evidence linking drug metabolism to the development of hypersensitivity is the detection of halothane‐specific antibodies in patients with liver failure^28^ and the observation that halothane protein adducts activate T cells.^29^ Furthermore, halothane derivatives that form lower levels of reactive metabolite are associated with a decreased risk of liver injury.^30^ To explore whether metabolites activate hypersensitive patient T cells in vitro*,* co‐culture strategies have been developed using microsomal metabolite generating systems.^31^, ^32^ Enhanced drug‐specific T‐cell responses have been reported with drugs in the presence of a metabolizing system; however, it is very difficult to delineate the disparate effects of the parent drug and metabolites. For this reason, we synthesized the reactive metabolite of sulfamethoxazole,^33^ which causes immune‐mediated skin and liver reactions that are not linked to expression of a specific HLA allele.^34^, ^35^ Working alongside other researchers in the field we have shown that PBMC and inflamed tissue from all hypersensitive patients contain T cells that are activated with sulfamethoxazole and/or its reactive metabolite via different pathways (ie, direct HLA binding and a hapten pathway, respectively).^12^, ^13^

In recent years, researchers studying hypersensitivity reactions strongly linked to expression of specific HLA alleles have focussed exclusively on the interaction between parent drug and the HLA molecule. This is because the parent drug is, for the most part, the only reagent available for in vitro cell culture studies. The observation that T cells from patients with hypersensitivity to drugs such as carbamazepine are activated with the parent drug^11^ has lead researchers to hypothesize that reactive metabolites are not involved in the development of HLA allele‐restricted forms of drug hypersensitivity. To investigate this hypothesis, we now focused on dapsone hypersensitivity in patients with leprosy. Dapsone is a model study drug as hypersensitivity reactions in patients are strongly linked to the expression of HLA‐B*13:01,^19^ the reactive nitroso metabolite of dapsone has been synthesized in a stable form,^15^ and patient samples are available for mechanistic studies. Utilizing PBMC from HLA‐B*13:01+ patients, we show CD4^+^ and CD8^+^ T‐cell responses to dapsone and the nitroso metabolite. Furthermore, dapsone and nitroso dapsone bind to HLA‐B*13:01 to selectively activate certain CD8^+^ clones.

Six HLA‐B*13:01+ patients that developed hypersensitivity while receiving dapsone in combination with rifampicin and clofazimine were recruited to the study. Three displayed a positive patch test response to dapsone. PBMC from all six patients were stimulated to proliferate and/or secrete IFN‐γ after stimulation with dapsone or the nitroso metabolite. The strongest in vitro responses were seen in the patients with the positive patch test response, and these patients were selected for serial dilution experiments to search for drug‐ and drug metabolite‐responsive T‐cell clones. CD4^+^ and CD8^+^ subpopulations were purified before plating single cells in culture plates for the cloning procedure. Dapsone‐ and nitroso dapsone‐responsive T cells were generated in almost equal numbers (dapsone 395; nitroso dapsone 399). The ratio of dapsone‐ or nitroso dapsone‐responsive CD4^+^ and CD8^+^ T‐cell clones was 4‐5:1 in patients 6 and 8 and 2:1 in patient 5, which demonstrates that drug exposure consistently results in CD4^+^ and CD8^+^ responses against the drug antigens. This is in stark contrast to the findings with abacavir where CD8^+^ T cells are the only cells activated with the drug.^36^, ^37^ The activation of CD4^+^ and CD8^+^ clones that expressed an array of TCR Vβ receptors, with dapsone or nitroso dapsone, was HLA class II and class I restricted, respectively. This confirms our previous data with PBMC from healthy donors,^15^ which shows that both dapsone and nitroso dapsone interact with HLA class I and class II molecules.

The availability of cloned CD4^+^ and CD8^+^ T cells permitted the detailed analysis of cross‐reactivity between dapsone, nitroso dapsone and structurally related compounds, mechanisms of dapsone‐ and nitroso dapsone‐specific T‐cell activation, and the involvement of the allele HLA‐B*13:01 in antigen presentation. Cross‐reactivity studies revealed that the position of the amine groups on the aromatic rings of dapsone and the availability of the sulfone group was critical for the activation of the T‐cell clones. Furthermore, as a panel of sulfonamides that contain one aromatic amine group did not activate the clones, both of dapsones’ aromatic amines were required for T‐cell activation.

25%‐50% of CD4^+^ and CD8^+^ clones were classified as highly specific as they were only activated with one compound (either the parent drug or metabolite [Figure 3B]). This confirms that T cells recognize and respond selectively to the two different forms of the dapsone antigen. Dapsone has an unusual structure in that the two aromatic amines connected to the sulfone group are identical. If the structure of a nitroso dapsone‐modified HLA binding peptide is compared with dapsone complexed to the same peptide, it is likely that similar conformations will be observed, and as such, the interaction with the T‐cell receptor will be the same. Thus, our working hypothesis to explain the drug‐ or drug metabolite‐specific activation of certain clones is that the HLA binding peptides also participate in the TCR interaction and impart a degree of selectivity.

When using optimum concentrations of dapsone (100‐500 µmol/L) and nitroso dapsone (5‐20 µmol/L) to activate CD4^+^ and CD8^+^ T cells, clones displaying high (ie, at least 50% of the response detected with the opposite compound) and low (ie, 10%‐50% of the response detected with the opposite compound) levels of cross‐reactivity were also detected. The majority of dapsone‐responsive, cross‐reactive CD4^+^ and CD8^+^ clones displayed low levels of cross‐reactivity with nitroso dapsone. Using quantitative mass spectrometry, we were able to demonstrate that the nitroso metabolite is reduced to dapsone in the 2‐day proliferation assay. Thus, clones incubated with 30 µmol/L nitroso dapsone were exposed to 2.5‐15 µmol/L of the parent drug, which stimulates a suboptimal T‐cell proliferative response. In contrast, the majority of cross‐reactive nitroso dapsone‐responsive CD4^+^ and CD8^+^ clones displayed high levels of cross‐reactivity with 100‐500 µmol/L dapsone. To investigate the response profiles further, a panel of cross‐reactive dapsone‐ and nitroso dapsone‐responsive clones were cultured with equal concentrations of the parent drug and metabolite (0.1‐100 µmol/L). Interestingly, weakly and strongly cross‐reactive clones were activated with the same concentrations of dapsone and nitroso dapsone with similar dose‐response curves until nitroso metabolite‐induced toxicity was detected. These data are in contrast to our earlier studies with sulfamethoxazole and its nitroso metabolite, where the parent drug activates T cells at significantly higher concentrations.^13^ It is reasonable to assume that HLA binding peptides play a less important role in the triggering of cross‐reactive TCR.

Given that clones displaying reactivity toward dapsone and/or the nitroso metabolite were detected, it was important to define mechanisms of antigen presentation including the requirement for antigen processing. Antigen‐presenting cells were required for the strong activation of CD4^+^ and CD8^+^ clones with dapsone and the nitroso metabolite. Clones were activated with dapsone in the presence of irradiated and glutaraldehyde‐fixed antigen‐presenting cells. In contrast, fixation reduced the strength of the nitroso dapsone‐specific T‐cell proliferative response. This suggests that dapsone activates T cells through a direct interaction with HLA molecules, whereas nitroso dapsone binds covalently to cellular protein forming adducts that require processing to generate antigenic peptides. As discussed above, the best explanation for the weak response of nitroso dapsone‐responsive clones with soluble nitroso dapsone and fixed antigen‐presenting cells is the formation of dapsone in the proliferation assay. The drug‐specific activation of clones from dapsone hypersensitive patients by two pathways has important implications for the future structural analysis of how dapsone and nitroso dapsone interacts with HLA molecules. Application of drug‐ or drug metabolite‐pulsed antigen‐presenting cells instead of soluble drug in T‐cell assays provides a tool to differentiate between the effects of covalent and non‐covalent forms of drug.^14^, ^39^ Dapsone‐responsive clones were not activated with dapsone‐pulsed antigen‐presenting cells. In contrast, 6 out of the 8 nitroso dapsone‐responsive clones tested were activated with nitroso dapsone‐pulsed antigen‐presenting cells and the strength of the induced response was the same as that seen with the parent drug. Two of the nitroso dapsone‐responsive clones were not activated with pulsed antigen‐presenting cells; interestingly, both of these clones cross‐reacted strongly with dapsone.

Glutathione is a tripeptide intracellular antioxidant that protects cells from exposure to aromatic nitroso compounds by acting as a reducing agent and through direct conjugation.^23^, ^24^ The addition of glutathione to T‐cell assays prevents the covalent binding of nitroso compounds, and hence, it is possible to explore whether T cells are activated with drug metabolite‐modified protein adducts.^40^ Nitroso dapsone glutathione adducts were formed rapidly in cultures containing the drug metabolite, T cells, antigen‐presenting cells, and glutathione. Moreover, glutathione decreased the response of clones to soluble nitroso dapsone and blocked the response to nitroso dapsone‐pulsed antigen‐presenting cells. Thus, the formation of a covalent adduct between nitroso dapsone and cellular proteins is needed for the activation of certain clones.

In the next section of the project, antigen‐presenting cells were generated from healthy donors expressing HLA‐B alleles with at least 90% sequence homology to HLA‐B*13:01 (including an additional HLA‐B*13:01+ donor) to investigate whether either dapsone or nitroso dapsone interact with a degree of selectivity to the risk allele to activate CD8^+^ clones. As described previously with other forms of drug hypersensitivity, several clones displayed HLA allele‐unrestricted drug recognition (ie, dapsone and nitroso dapsone stimulated a proliferative response in the presence of multiple antigen‐presenting cells expressing different HLA‐B alleles).^25^ Other clones were only activated in the presence of nitroso dapsone and autologous antigen‐presenting cells. However, a panel of clones were selectively activated with dapsone or nitroso dapsone in the presence of antigen‐presenting cells displaying HLA‐B*13:01. Cross‐reactivity was observed for several of these clones; however, no predominant TCR Vβ receptors were expressed. This indicates that both the parent drug and reactive metabolite interacts with HLA‐B*13:01 to activate certain CD8^+^ clones. We are therefore synthesizing designer nitroso dapsone‐modified HLA‐B*13:01 binding peptides and conducting structural analyses to compare the binding interaction of dapsone and nitroso dapsone with HLA‐B*13:01.

Different forms of drug hypersensitivity reaction are associated with the secretion of a diverse panel of cytokines from T cells.^41^ Thus, ELIspot was used to profile cytokine secretion from dapsone‐ and nitroso dapsone‐stimulated clones. CD4^+^ and CD8^+^ clones secreted the same panel of cytokines when stimulated with dapsone or nitroso dapsone, but expressed distinct chemokine receptors. Clones secreted Th1, Th2, and Th22 cytokines alongside the cytolytic molecules perforin, granzyme B, and FasL; however, IL‐17 was not detected. IL‐22 secretion in the absence of IL‐17 seems to be a common feature of drug‐specific clones as this profile has now been detected with dapsone, sulfamethoxazole, and piperacillin.^42^, ^43^ It is possible that this profile relates to the long‐term in vitro culture of clones; thus, a future study exploring the cytokine profile in affected tissues would be appropriate. The chemokine receptors displayed on dapsone‐ or nitroso dapsone‐responsive CD4^+^ clones were restricted to CXCR3 and CCR4. In contrast, CD8^+^ clones also expressed CCR6, 9, and 10. CCR4 and CCR10 have been implicated in the migration of T cells into skin^44^; thus, a more detailed investigation of migratory properties of CD4^+^ and CD8^+^ T cells in patients with dapsone hypersensitivity is warranted.

In conclusion, our study shows that dapsone‐ and nitroso dapsone‐responsive CD4^+^ and CD8^+^ T cells circulate in hypersensitive patients. T cells were activated selectively with the parent drug and drug metabolite via direct HLA binding and a hapten mechanism, respectively. The detection of HLA‐B*13:01‐restricted dapsone‐ and nitroso dapsone‐responsive CD8^+^ clones indicates that dapsone hypersensitivity should be used as an exemplar to explore the structural features of drug HLA binding and how this interaction results in a pathogenic T‐cell response. For patients with suspected hypersensitivity, it is critical to identify the agent that caused the reaction. We are currently exploring whether drug‐peptide antigens represent effective reagents for T‐cell activation. A better understanding of drug antigenicity will lead to the development of improved evidence‐based diagnostic tools.

## CONFLICTS OF INTEREST

The authors declare that they have no conflicts of interest.

## AUTHOR CONTRIBUTIONS

QZ, KA, AA, MA, JA, AG, and MOO conducted the biological experiments. XM conducted the mass spectrometric analyses. HL, LD, and HZ collected the clinical samples and conducted patch testing. DJN, BKP, DAO, JL, and FZ designed the study and supervised the project. AG, MOO, XM, and DJN analyzed the data and drafted the manuscript. All authors critically reviewed the manuscript.
