# Supplementary figures and images for: Dapsone‐ and nitroso dapsone‐specific activation of T cells from hypersensitive patients expressing the risk allele HLA‐B*13:01
Source: Allergy. 2019 Apr 15;74(8):1533–48. doi: 10.1111/all.13769 (PMC6767778; doi:10.1111/all.13769)

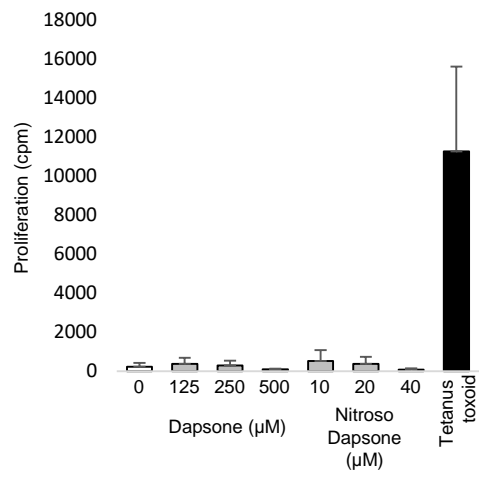

Supplement: Supplementary file 1 [file ALL-74-1533-s001.pdf]

*CD4+ clones*

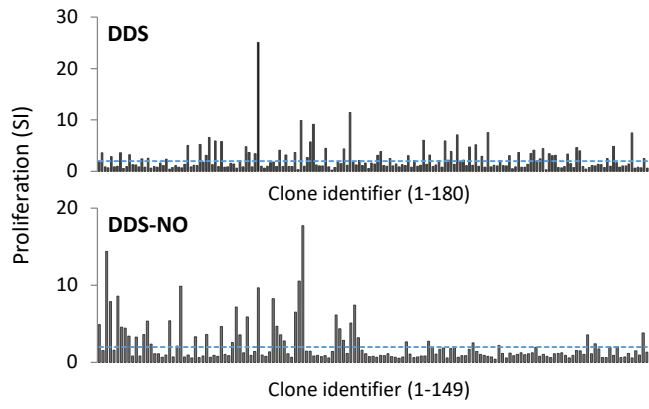

Patient 5

*CD8+ clones*

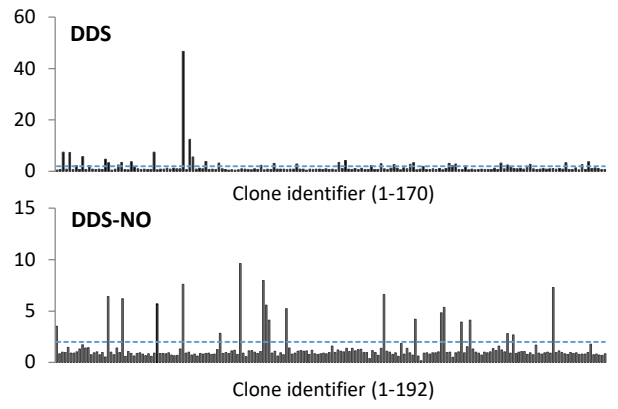

Patient 6

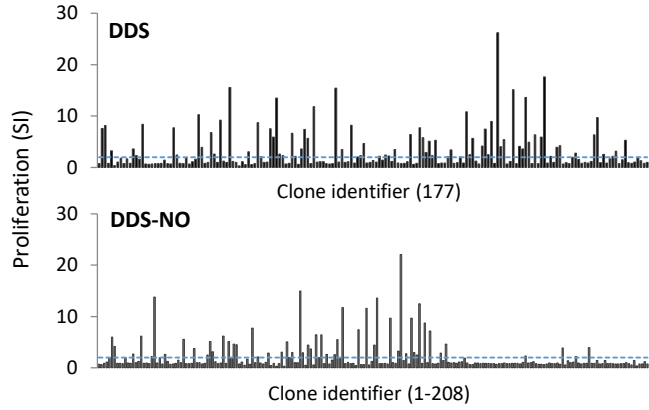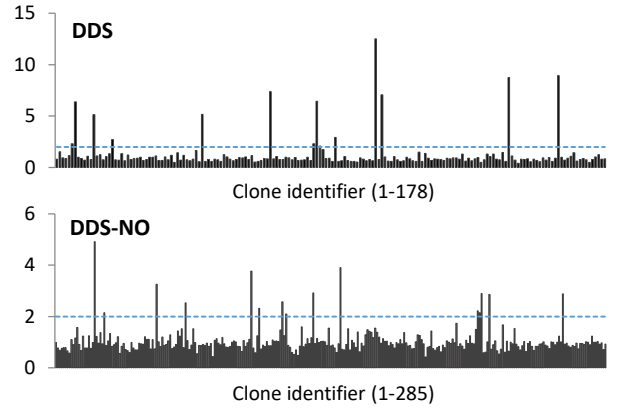

Patient 8

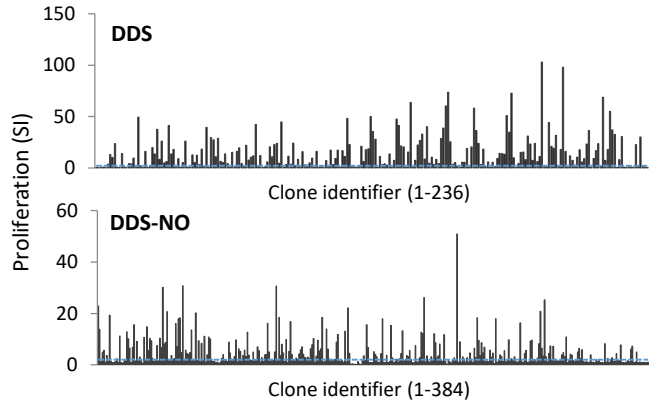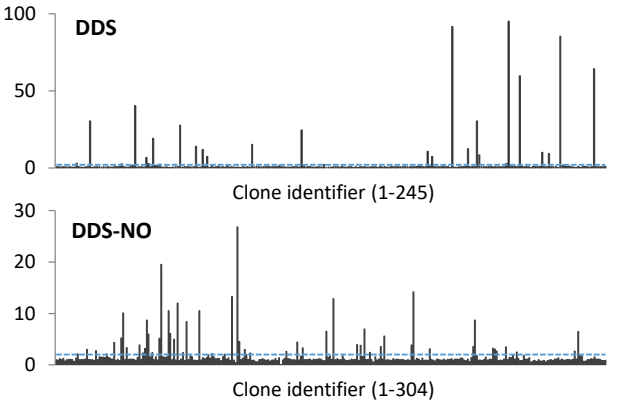

Supplement: Supplementary file 2 [file ALL-74-1533-s002.pdf]

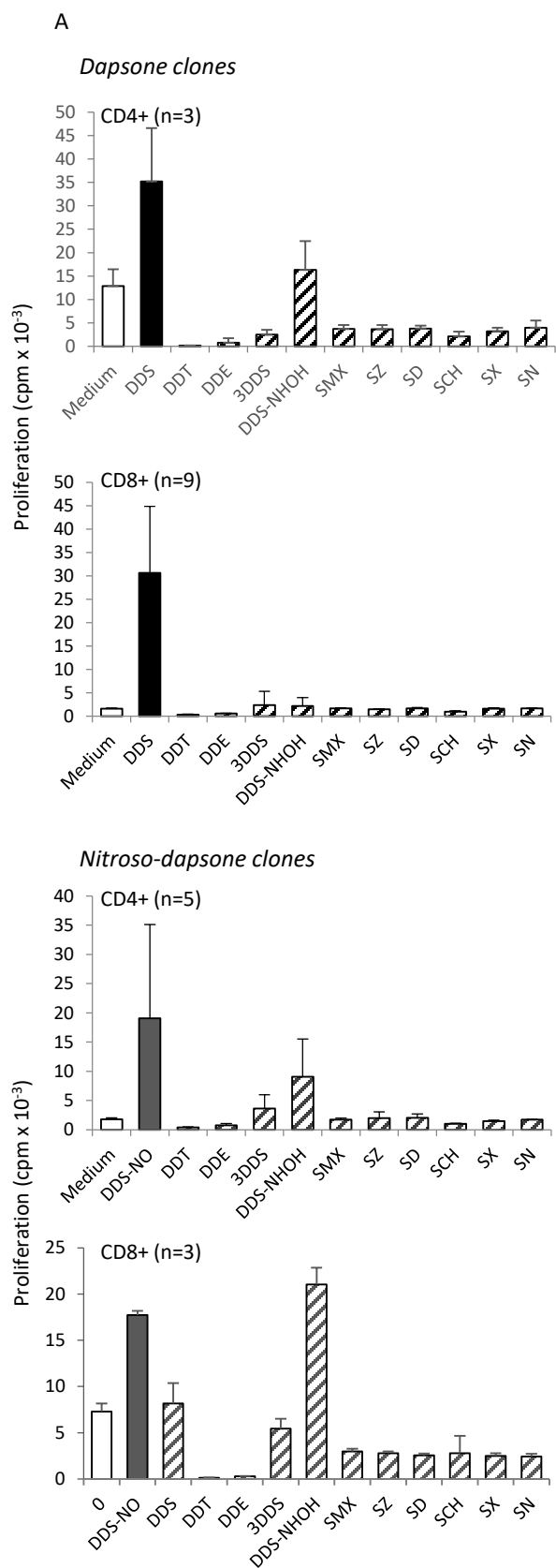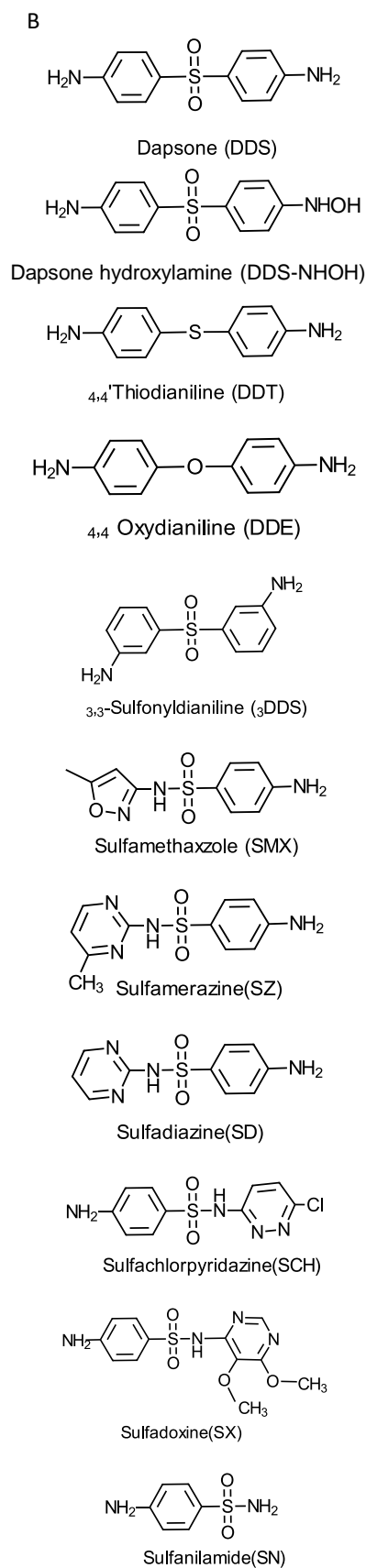

Supplement: Supplementary file 3 [file ALL-74-1533-s003.pdf]

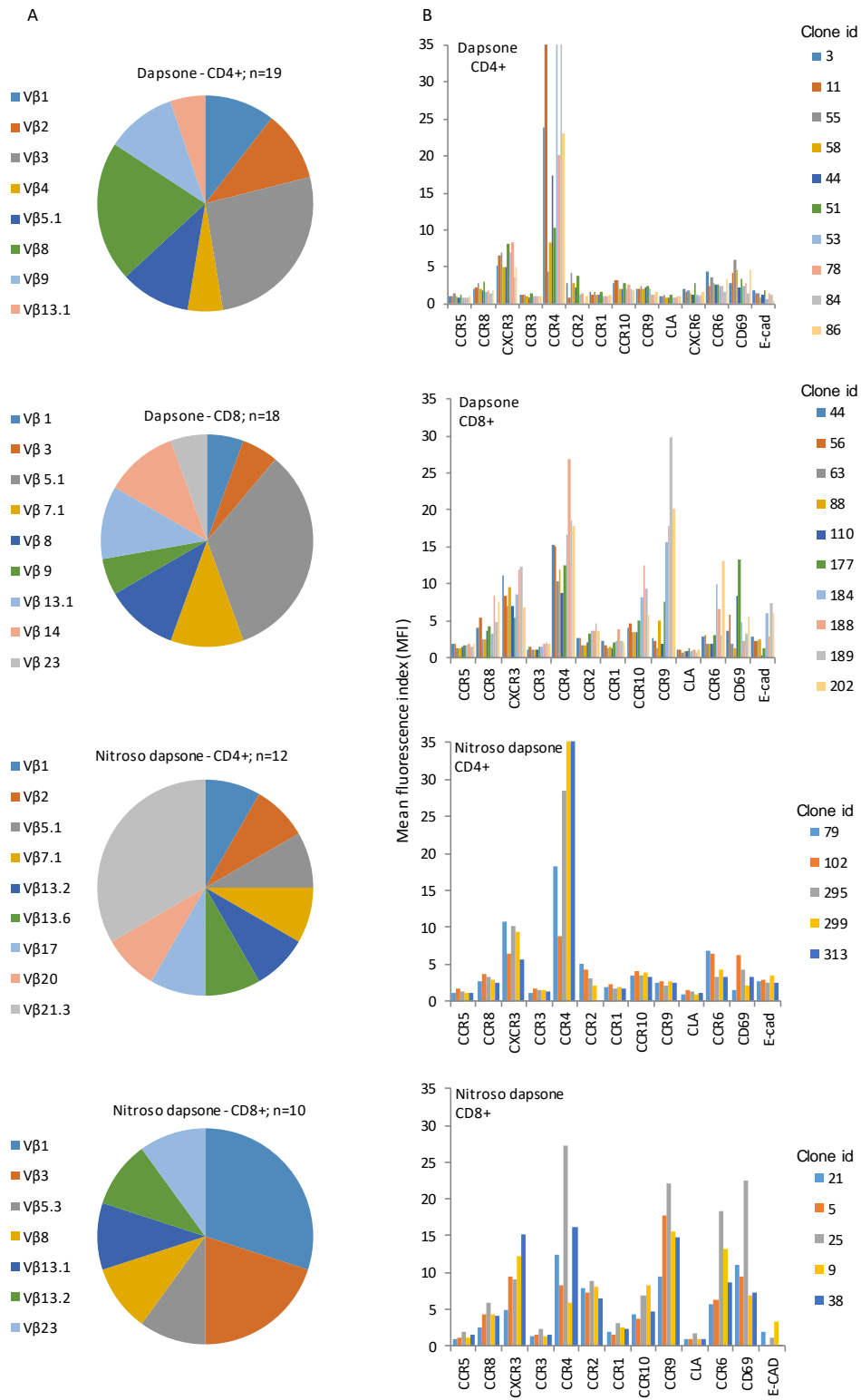

**C**

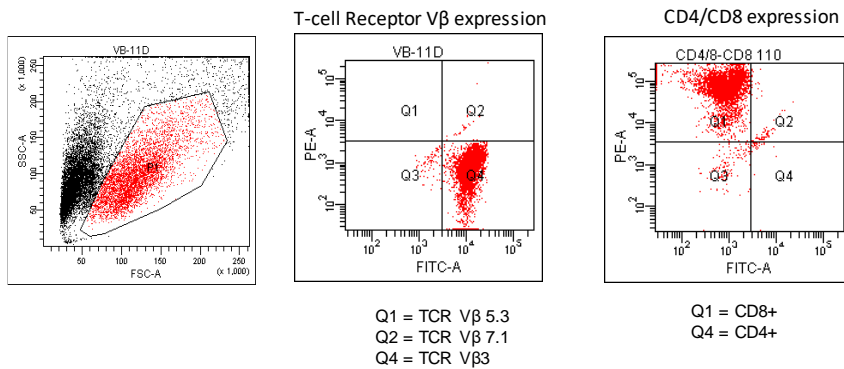

Supplement: Supplementary file 4 [file ALL-74-1533-s004.pdf]

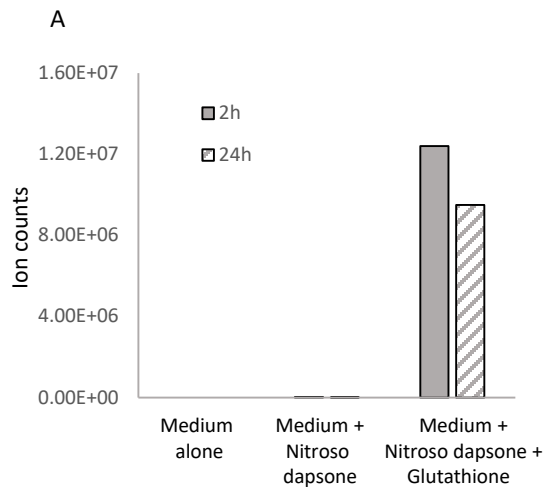

*Nitroso-dapsone clones*

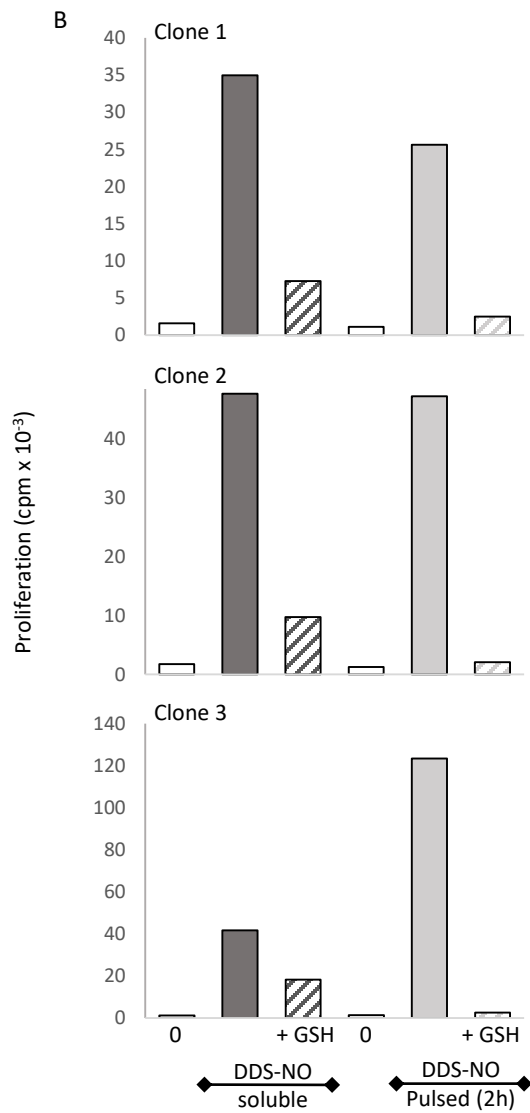

Supplement: Supplementary file 5 [file ALL-74-1533-s005.pdf]

A.

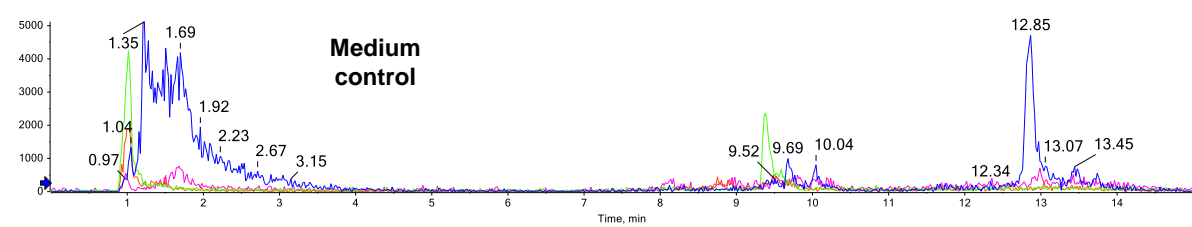

B.

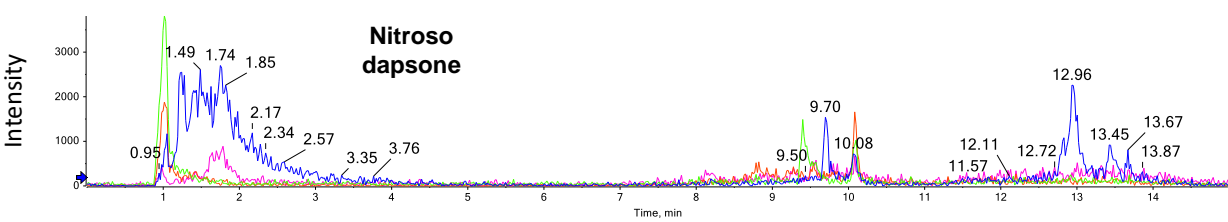

C.

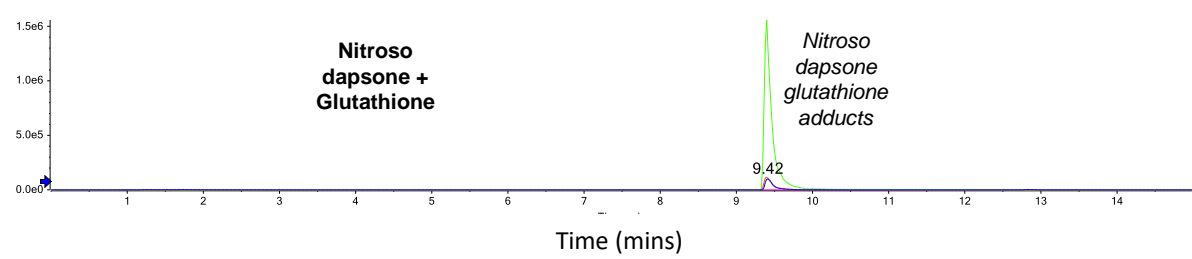

Supplement: Supplementary file 6 [file ALL-74-1533-s006.pdf]
