# Supplementary material for: Dapsone‐ and nitroso dapsone‐specific activation of T cells from hypersensitive patients expressing the risk allele HLA‐B*13:01
Source: Allergy. 2019 Apr 15;74(8):1533–48. doi: 10.1111/all.13769 (PMC6767778; doi:10.1111/all.13769)
Supplement: Supplementary file 7 [file ALL-74-1533-s007.docx]

**Supplementary Text**

**Generation of drug-specific T-cell clones**

PBMC (1-5x10^6^/ml) from hypersensitive patients 5, 6 and 8 were incubated with dapsone (125-250µM) and nitroso dapsone (10-20µM) in IL-2 (100IU/ml) containing medium to generate T-cell lines. Culture medium consisted of RPMI-1640 supplemented with pooled heat-inactivated human AB serum (10%, v/v), HEPES (25mM), L-glutamine (2mM), transferrin (25μg/mL), streptomycin (100μg/mL), and penicillin (100U/mL). After 14 days, T-cells were serially diluted (0.3-3 cells/well), and subjected to PHA-driven expansion (5µg/ml). Irradiated allogeneic PBMC (5x10^4^/well) were added as feeder cells. After 28 days, clones expanded to approximately 5x10^5^ cells were tested for drug specificity by culturing dapsone (250µM; 200µl total volume) or nitroso dapsone (20µM) with clones (5x10^4^ cells/well) and irradiated Epstein-Barr virus (EBV)-transformed B-cells (1x10^4^ cells/well) for 48h in triplicate cultures per experimental condition. EBV-transformed B-cell lines were generated from autologous PBMC by culturing the PBMC with supernatant from the B9-58 cell line containing cyclosporine A. The EBV-transformed B-cell lines were used as antigen presenting cells. Proliferation was measured by the addition of [^3^H]thymidine for 16h followed by scintillation counting. Clones with a stimulation index (SI) (mean cpm drug-treated wells / mean cpm in control wells) of greater than 2 were expanded and analysed further.

**Phenotype and specificity testing of drug-specific T-cell clones**

Cell phenotyping was performed by flow cytometry. TCR Vβ expression was measured using the IOTest® Beta Mark, TCR Vβ Repertoire Kit (Beckman Coulter). Antibodies used for flow cytometry staining purchased from BD Biosciences (Oxford, UK) were CD4-APC (clone RPA T4), CD8-PE (clone HIT8a), CCR4-PE (clone 1G1), CLA-FITC (clone HECA-452); and from R&D Systems (Abingdon, UK) were CCR1-Alex Fluor 488 (clone 53504), CCR2-PE (clone 48607), CCR3-FITC (clone 61828), CCR5-FITC (clone CTC5), CCR6-APC (Clone 53103), CCR8-PE (clone 191704),  CCR9-APC (clone 248621), CCR10-PE (clone 314305), CXCR1-FITC (clone 42705), CXCR3-APC (clone 49801), CXCR6-PE (clone 56811) and E cadherin-Alexa Fluor 488 (clone 180224). Approximately 1x10^5^ T cell clones were stained for surface markers using directly-conjugated antibodies. The cells were incubated on ice for 20 min and then washed with 1ml 10% foetal calf serum in Hanks balanced salt solution. Chemokine receptor expression is presented as mean fluorescence intensity of the whole population of each clone. All cells were acquired using a FACS Canto II (BD Biosciences, Oxford, UK) and data analyzed by Cyflogic. A minimum of 50,000 cells were acquired using FSC/SSC characteristics.

Dose-dependent proliferative responses and the profile of secreted cytokines (IFN-γ, IL-5, IL-13, granzyme B, Fas L, perforin, IL-17 and IL22) from CD4+ and CD8+ clones were measured using [^3^H]thymidine and ELIspot, respectively. The ELIspot reader accurately counts spots up to approximately 400-500; thus, giving an upper limit to the assay. In both experiments T-cell clones (5x10^4^) were cultured with irradiated antigen presenting cells (1x10^4^) and dapsone (0.01-500µM) or nitroso dapsone (0.01-100µM; 200µl) for 48 h.

Dapsone- and nitroso dapsone-responsive clones (5x10^4^) were also cultured with non-toxic concentrations of structurally-related compounds (sulfamethoxazole, sulfamerazine, sulfadiazine, sulfachloropyridazine, sulfadoxin, sulphanilamide, 4,4 thiodianiline, 4,4 oxyaniline, 3,3 sulfonyldianiline and mono and diacetylated forms of dapsone) and antigen presenting cells (1x10^4^; 200µl) for 48h. Proliferation was measured by the addition of [^3^H]thymidine followed by scintillation counting.

**Pathways of drug presentation to T-cells**

To explore the pathway of CD4+ and CD8+ T-cell activation with dapsone and nitroso dapsone, T-cell clones were subjected to a variety of experimental protocols. First, clones were cultured with optimal concentrations of dapsone or nitroso dapsone in the presence or absence of antigen presenting cells. Second, clones were cultured with dapsone or nitroso dapsone and antigen presenting cells in the presence or absence of isotype (IgG1) or MHC class I (DX17) and class II (Tu39) blocking antibodies were purchased from BD Biosciences. Third, clones were cultured with dapsone or nitroso dapsone and glutaraldehyde-fixed antigen presenting cells. Fixation blocks protein processing. Briefly, EBV-transformed B-cells (2×10^6^ cells/ml) were washed in HBSS buffer to exclude FBS and re-suspended in HBSS buffer (1 ml). Glutaraldehyde (25%, 1 µL) was added to the cells and gently mixed for 30 secs. Glycine (1ml of 1 M) was quickly added to the cell suspension and mixed for a further 45 secs. Cells were washed three times to remove glutaraldehyde and then resuspend in T-lymphocyte culture medium. This was followed by co-culture of drug-specific T-cell clones (5×10^4^, 50 µL) with glutaraldehyde-fixed EBV-transformed B-cells (1×10^4^ cells, 50 µL) in the presence or absence of the drug antigen (100 µL) in a 96-well U-bottom microplate for 48 hrs, 5% CO_2_ at 37˚C. [^3^H]-thymidine was added for the final 16 hours of incubation to evaluate T-lymphocyte proliferation. Fourth, antigen presenting cells pulsed with dapsone or nitroso dapsone for 0.5-2h were used to activate T-cells in the absence of soluble drug. Fifth, clones were cultured with dapsone or nitroso dapsone and antigen presenting cells in the presence or absence of glutathione (1mM). Glutathione binds covalently to aromatic nitroso compounds, limiting their protein reactivity (24). The stability of dapsone and nitroso dapsone and the formation of dapsone glutathione adducts during the culture period were measured by mass spectrometry. Briefly, aliquots of cell culture supernatant (20 µL) and the calibration standards (20 µL) were diluted with LC-MS grade water (1:10 dilution) and deproteinized with acetonitrile. The extracts were evaporated to dryness in a Speed Vac and reconstituted in 200 µL water. 5 µL samples and standards were analysed immediately by a Triple Quad^TM^ 6500 mass spectrometer (AB Sciex,) coupled with a 1260 Infinity LC system (Agilent Technologies, Germany). The multiple reaction monitoring transitions for each analyte were as following: dapsone 249.1/156.1 and 249.1/107.9; azoxy dapsone, 509.1/108.1 and 509.1/156.1; dapsone-GSH (3O), 602.1/401.3, 602.1/156.1, and 602.1/261.5. Other MS parameters, such as voltage potential and collision energy were optimised to achieve greatest sensitivity. Data acquisition and quantification were performed using Analyst 1.5 software and Multi-Quant 3.0 (AB Sciex).

**The involvement of HLA-B*13:01 in the activation of CD8+ T-cell clones**

To explore whether dapsone and nitroso dapsone interact with HLA-B*13:01 to activate CD8+ clones, EBV-transformed B-cells were generated from 9 healthy donors expressing HLA alleles displaying at least 90% similarity to HLA-B*13:01 (Supplementary Table 2). EBV-transformed B-cells from one additional hypersensitive patient expressing HLA-B*13:01 itself were also used (Supplementary Table 3). CD8+ T-cell clones were cultured with the different antigen presenting cells and dapsone or nitroso dapsone for 48h. Proliferation was measured by the addition of [^3^H]thymidine followed by scintillation counting.

**Supplementary Table 1. HLA typing of hypersensitive patients**

|  | HLA-A^1^ | | HLA-B | | HLA-C | | HLA-DQB1 | | HLA-DRB1 | |
| --- | --- | --- | --- | --- | --- | --- | --- | --- | --- | --- |
| Patient 1 | 02:07 | 24:02 | *13:01* | 40:01 | 03:04 | 15:02 | 03:01 | 06:01 | 11:01 | 15:01 |
| Patient 5 | 02:07 | 11:01 | *13:01* | 40:01 | 03:04 | 07:02 | 06:01 | 06:01 | 08:03 | 15:01 |
| Patient 6 | 02:07 | 11:01 | *13:01* | 46:01 | 01:02 | 03:04 | 06:01 | 06:10 | 15:01 | 15:01 |
| Patient 7 | 11:01 | 11:01 | *13:01* | 15:32 | 03:04 | 12:03 | 03:01 | 03:01 | 11:06 | 13:12 |
| Patient 8 | 11:01 | 24:02 | *13:01* | 15:25 | 03:04 | 04:03 | 03:01 | 05:02 | 12:02 | 15:01 |

^1^ Patient 3 PBMC were not available for HLA typing

**Supplementary Table 2. HLA typing of antigen presenting cells from healthy donors**

| **Alleles with ↑ 90% amino acid B*13:01 match** | **Sequence similarity** | **HLA alleles expressed on antigen presenting cells from healthy donors** | | | | | | | | | | | | | |
| --- | --- | --- | --- | --- | --- | --- | --- | --- | --- | --- | --- | --- | --- | --- | --- |
|  |  | **HLA-A** | | **HLA-B** | | **HLA-C** | | | **HLA-DRB1** | | **HLA-DQB1** | | **HLA-DQA1** | | |
| **Donor 1 (B*08:01)** | 91.7 | 01:01 | 01:01 | *08:01* | *57:01* | 07:01 | | 07:01 | 04:04 | 07:01 | 03:03 | 04:02 | 02:01 | | 03:01 |
| **Donor 2 (B*13:02)** | 99.2 | 02:01 | 30:01 | *13:02* | *57:01* | 06:02 | | 7:01 | 07:01 | 07:01 | 02:01 | 03:03 | 02:01 | | 02:01 |
| **Donor 3 (B*15:01)** | 93.9 | 01:01 | 03:01 | *15:01* | *57:01* | 03:03 | | 6:02 | 04:01 | 07:01 | 03:02 | 03:03 | 02:01 | | 03:01 |
| **Donor 4 (B*35:01)** | 94.5 | 03:01 | 26:01 | *35:01* | *35:01* | 04:01 | | 4:01 | 01:01 | 11:01 | 03:01 | 05:01 | 01:01 | | 05:01 |
| **Donor 5 (B*40:01)** | 93.4 | 02:01 | 03:02 | *40:01* | *51:01* | 03:04 | | 4:02 | 11:04 | 11:01 | 03:01 | 03:01 | 05:01 | | 05:01 |
| **Donor 6 (B*44:02)** | 96.7 | 02:01 | 02:01 | *44:02* | *57:01* | 05:01 | | 06:02 | *04:01 | 07:01 | 03:01 | 03:03 | 02:01 | | 03:01 |
| **Donor 7 (B*46:01)** | 92.3 | 11:01 | 24:02 | *46:01* | *58:01* | 01:02 | | 03:02 | 03:01 | 09:01 | 02:01 | 03:03 | 03:01 | | 05:01 |
| **Donor 8 (B*51:01)** | 94.2 | 11:01 | 11:01 | *51:01* | *52:04* | 04:01 | | 12:02 | 04:02 | 14:04 | 03:02 | 05:03 | 01:01 | | 03:01 |
| **Donor 9 (B*58:01)** | 94.2 | 02:06 | 02:01 | *15:01* | *58:01* | 01:02 | 07:01 | | 13:02 | 15:01 | 06:09 | 06:02 | | 01:02 | 01:02 |
| **Donor 10^1^ (B*13:01)** | 100 |  |  | *13:01* |  |  |  | |  |  |  |  | |  |  |

^1^ Antigen presenting cells from hypersensitive patients 5, 6 or 8 were used. See Table E2 for HLA typing data.

**Supplementary Table 3. Cytokine secretion from dapsone and nitroso dapsone-responsive CD4+ and CD8+ T-cell clones.**

|  |  | **IFN-γ** | **IL-5** | **IL-13** | **IL-17** | **IL-22** | **Per-forin** | **Granz-yme B** | **Fas L** |
| --- | --- | --- | --- | --- | --- | --- | --- | --- | --- |
| CD4 | DDS | 100^1^ | 88 | 82 | 0 | 100 | 94 | 94 | 92 |
|  | DDS-NO | 80 | 100 | 60 | 0 | 100 | 60 | 80 | 92 |
| CD8 | DDS | 100 | 79 | 57 | 0 | 78 | 93 | 78 | 100 |
|  | DDS-NO | 100 | 100 | 100 | 0 | 90 | 100 | 100 | 100 |

^1^ Numbers refer to the percentage of clones with an increase of at least 50 spot forming units (sfu) when drug (DDS, dapsone; DDS-NO, nitroso dapsone)- and vehicle-treated wells were compared.
